# A Pilot Study of Mindfulness-Based Exposure Therapy in OEF/OIF Combat Veterans with PTSD: Altered Medial Frontal Cortex and Amygdala Responses in Social–Emotional Processing

**DOI:** 10.3389/fpsyt.2016.00154

**Published:** 2016-09-20

**Authors:** Anthony P. King, Stefanie R. Block, Rebecca K. Sripada, Sheila A. M. Rauch, Katherine E. Porter, Todd K. Favorite, Nicholas Giardino, Israel Liberzon

**Affiliations:** ^1^Mental Health Service, VA Ann Arbor Health System, Ann Arbor, MI, USA; ^2^Department of Psychiatry, University of Michigan, Ann Arbor, MI, USA; ^3^Department of Psychology, University of Michigan, Ann Arbor, MI, USA; ^4^VA Center for Clinical Management Research, Ann Arbor, MI, USA; ^5^Department of Psychiatry and Behavioral Sciences, Emory University, Atlanta, GA, USA; ^6^Mental Health Service, Atlanta VA Medical Center, Atlanta, GA, USA; ^7^Mary A. Rackham Institute (MARI), University of Michigan, Ann Arbor, MI, USA

**Keywords:** PTSD, mindfulness, fMRI BOLD, amygdala, exposure therapy, dorsal anterior cingulate cortex

## Abstract

Combat-related posttraumatic stress disorder (PTSD) is common among returning veterans, and is a serious and debilitating disorder. While highly effective treatments involving trauma exposure exist, difficulties with engagement and early drop may lead to sub-optimal outcomes. Mindfulness training may provide a method for increasing emotional regulation skills that may improve engagement in trauma-focused therapy. Here, we examine potential neural correlates of mindfulness training and *in vivo* exposure (non-trauma focused) using a novel group therapy [mindfulness-based exposure therapy (MBET)] in Afghanistan (OEF) or Iraq (OIF) combat veterans with PTSD. OEF/OIF combat veterans with PTSD (*N* = 23) were treated with MBET (*N* = 14) or a comparison group therapy [Present-centered group therapy (PCGT), *N* = 9]. PTSD symptoms were assessed at pre- and post-therapy with Clinician Administered PTSD scale. Functional neuroimaging (3-T fMRI) before and after therapy examined responses to emotional faces (angry, fearful, and neutral faces). Patients treated with MBET had reduced PTSD symptoms (effect size *d* = 0.92) but effect was not significantly different from PCGT (*d* = 0.43). Improvement in PTSD symptoms from pre- to post-treatment in both treatment groups was correlated with increased activity in rostral anterior cingulate cortex, dorsal medial prefrontal cortex (mPFC), and left amygdala. The MBET group showed greater increases in amygdala and fusiform gyrus responses to Angry faces, as well as increased response in left mPFC to Fearful faces. These preliminary findings provide intriguing evidence that MBET group therapy for PTSD may lead to changes in neural processing of social–emotional threat related to symptom reduction.

## Introduction

Posttraumatic stress disorder (PTSD) affects up to ~20% of combat veterans returning from Afghanistan (OEF) and Iraq (OIF) (1), and is associated with high levels of suffering and disability. There is considerable empirical support for exposure-based individual psychotherapy for PTSD involving processing of traumatic memories [e.g. prolonged exposure therapy (PE), cognitive processing therapy (CPT)] and these individual treatments show very large effect sizes in military veterans compared to baseline (pre–post Cohen’s *d* in the 1.0–2.0 range) and often in comparison to active therapies (2, 3). However, even with the strong evidence of efficacy for exposure therapy, many veterans with PTSD do not fully engage in treatment and others drop-out prior to receiving full benefit (3, 4).

Group psychotherapy is a common modality in PTSD treatment, in particular in the veterans affairs (VA) health system in the United States, and recent data suggest that they may be associated with better adherence and retention compared to individual therapies (5). It would appear that group therapies might provide a useful way to deliver non-trauma-focused self-regulation, coping skills, and social skills, because they capitalize on patients’ collaborative support in a group environment and may be efficient. However, group therapies appear to be considerably less effective than individual therapy for PTSD symptom reduction, even when the content of the intervention was similar [e.g., individual vs. group present-centered therapy (PCT), trauma-focused exposure therapy, CPT] (6–8). A meta-analysis of group therapy for PTSD reported an overall mean effect size of *d* = 0.56 in all populations (9), with lower efficacy in men and in particular, considerably lower efficacy in male combat veterans with PTSD (effect sizes ranging from 0.09 to 0.31). A recent large study of women veterans (*N* = 272) treated with group form of CPT that was not included in the meta-analysis found pre-post effect size estimate range of 0.27–0.38 (8).

Mindfulness-based interventions, such as mindfulness-based stress reduction (MBSR) and mindfulness-based cognitive therapy (MBCT), involve training in attention and emotional regulation (10, 11) and are delivered in group format. There is accumulating evidence that MBCT and MBSR may be helpful for depression, and anxiety: MBCT has demonstrated effectiveness for relapse prevention in patients with severe, chronic major depressive disorder (12), and MBSR and MBCT appear useful for depression (13, 14) and generalized anxiety disorder (GAD) (15). Recent studies have suggested that mindfulness training may also be a useful component for treatment of PTSD. Improvement in depression and PTSD symptoms has been shown in pilot studies of MBSR in adults with childhood sexual abuse history (16, 17) and combat veterans (18, 19). We found that an 8-week MBCT specifically adapted for PTSD led to improvement in avoidant symptoms in chronic PTSD patients (Vietnam veterans) (20), and recent larger randomized controlled studies of MBSR adapted for combat PTSD in primarily Vietnam veterans have shown similar results (21).

We have been interested to examine whether mindfulness training together with non-trauma-focused *in vivo* exposure may provide emotion regulation skills in a group modality that might lead to improvement in PTSD symptoms, as well as increased engagement and retention in subsequent individual trauma-focused therapy. We also wanted to examine whether mindfulness training in the context of *in vivo* exposure might be associated with unique neural signatures. There has been considerable amount of neuroimaging research on the emotional neurocircuitry of PTSD [meta-analyzed in (22–25)], which has implicated increased activity in amygdala, insula, and mid- and dorsal anterior cingulate cortex (ACC) in trauma recall (e.g., trauma script-driven imagery, visual and auditory presentation of trauma-related cues), and non-trauma-related aversive social–emotional processing (visual presentation of fearful and angry emotional faces and aversive social scenes) and decreased activity in frontal lobe regions (e.g., ventral medial prefrontal cortex (mPFC), rostral ACC, and some regions of dorsal ACC) in trauma recall, non-trauma emotional stimuli, and emotional Stroop tasks in PTSD. However, there have been relatively few neuroimaging studies of PTSD psychotherapy. Studies of neural predictors of treatment outcome have reported better therapy responses associated with lower levels of pre-therapy amygdala and rostral ACC responses to rapidly presented fearful faces (26, 27) and higher dorsal striatal and frontal activations during inhibitory tasks (28). A small number of longitudinal (“pre–post” design) studies have reported changes in brain activations that differ between treatment responders and non-responders, including ACC and insula activation on tasks involving anticipation of negative or positive images (29, 30) and Stroop task (31), as well as changes in left inferior parietal lobe (IPL) during contextual cue processing (32).

Several imaging studies have shown alterations in resting-state functional connectivity (rsFC) in long-term meditators (33–36). Similar effects on rsFC have also been reported in healthy persons and patients randomized to brief (6–8 week) group meditation trainings (37–41), and changes in interoceptive processes have also been reported (42, 43). A small number of neuroimaging studies of mindfulness training in anxiety disorder patients have found MBSR-related changes in self-related neural processing in social anxiety (44, 45), and dorsolateral PFC and amygdala–frontal cortex connectivity in GAD (46) associated with symptom improvement, i.e., similar regions as implicated in improvement in PTSD symptoms. We recently conducted a pilot trial of a novel non-trauma-focused PTSD group intervention incorporating PTSD psycho-education, mindfulness training, and *in vivo* exposure, “mindfulness-based exposure therapy” (MBET), compared to a manualized comparison group therapy, present-centered group therapy [PCGT, originally designed as a control for exposure-based therapies (7, 47)]. The present study examines effects of MBET (which contains both mindfulness training and *in vivo* exposure) on neural correlates of PTSD symptom reduction, in a pre–post therapy fMRI study using a probe of social–emotional threat, presentation of faces with angry, fearful, and neutral expressions. Based on previous findings of decreased activity in mPFC/ACC during emotional tasks in PTSD patients (22–25), we hypothesized that improvement in PTSD symptoms would be associated with increased activity in medial frontal cortex when viewing faces with threatening emotional expressions. Given the mixed findings of altered amygdala activity during emotional processing in PTSD, we hypothesized changed activity in these regions.

## Materials and Methods

### Methods

#### Participants

The study was approved by the Institutional Review Boards at the University of Michigan Medical School and the Ann Arbor VA. Written informed consent was obtained after a complete description of the study was provided to the participants. We recruited male OEF/OIF combat veterans with PTSD (*N* = 23) seeking treatment for PTSD at the VA Ann Arbor Health System (see Table 1). All individuals met DSM-IV criteria for current (past month) PTSD, as assessed by the Clinician Administered PTSD Scale (CAPS) (38). Participants were also assessed for comorbid disorders using the Mini-International Neuropsychiatric Interview (MINI) (39). Participants with psychosis, personality disorders, or suicidal risk were excluded. Psychiatric medications were allowed, but no changes in medications were allowed from 4 weeks before the intake scan until the end of the interventions and post-intervention scan. No differences in presence of medications were observed between the treatment group (medications included SSRI: citalopram, fluoxetine, sertraline, venlafaxine; Sleep: trazadone, zolpidem; Pain: codeine, morphine, tramadol; Benzodiazepine: clonazepam, lorazepam; Other: aripiprazole, buproprion, prazocin, sumatriptan, topiramate).

**Table 1:** **Demographics**.

Characteristic	MBET (*N* = 14)	PCGT (*N* = 9)	*t/*χ^2^	*p*
Age, M (SD)	32.43 (7.54)	31.67 (10.14)	0.207	0.838
Race, *N* (%)			0.109	0.742
European American	13 (93%)	8 (89%)		
African American	1 (7%)	1 (11%)		
Education, *N* (%)			1.431	0.489
Some grad school or graduate degree	1	1		
Some college or college degree	11	5		
High school grad	2	3		
CAPS, M (SD)	72.29 (18.32)	74.11 (15.34)	0.213	0.833
Comorbidities, *N* (%)				
Mood disorder	13 (93%)	6 (67%)	2.616	0.106
Anxiety disorder	3 (21%)	1 (11%)	0.406	0.524
Substance use disorder	3 (21%)	0	2.218	0.136
Psychiatric medications				
None	2 (14%)	3 (33%)	1.168	0.280
SSRI	4 (29%)	3 (33%)	0.059	0.809
Sleep	2 (14%)	2 (22%)	0.240	0.624
Benzodiazepine	2 (21%)	1 (11%)	0.406	0.524
Pain	2 (21%)	1 (11%)	0.406	0.524
Other	5 (36%)	2 (22%)	1.028	0.311

#### Procedure

Participants underwent a functional magnetic resonance imaging (fMRI) scan within 2 weeks of the initial screening visit. Patients were randomized into one of two 16-week group therapy groups for PTSD: MBET or PCGT, each with two to six patients per group. However, randomization was only partial because the PCGT arm was discontinued during the trial due to higher drop-out rate and recruitment difficulties. Within 2 weeks after the end of the interventions, participants underwent a second diagnostic interview and fMRI scan.

#### Therapists and Raters

Therapists were doctoral and masters-level psychologists at a VA PTSD clinic who had training in MBCT and in PCT. Trained, experienced clinical raters administered diagnostic clinical interviews (CAPS and MINI) and were blind to treatment condition.

##### Mindfulness-Based Exposure Therapy

This 16-week non-trauma-focused intervention was developed by the authors at the VA Ann Arbor, incorporating mindfulness training from MBCT, PTSD psycho-education and *in vivo* exposure from prolonged exposure therapy, and self-compassion exercises as we have recently described (48, 49). *In vivo* exposures were conducted only to avoided (and objectively safe) situations or activities. It was explicitly stated that no imaginal exposure or processing of trauma memories would be done in this group. The intervention consists of four “modules”: (a) PTSD psycho-education and relaxation, (b) mindfulness of body and breath exercises and *in vivo* exposure, (c) mindfulness of emotion and *in vivo* exposure, and (d) self-compassion training. The group sessions were 2 h each and participants completed daily homework in between sessions, involving formal and informal mindfulness practice and *in vivo* exposure, but no trauma exposure or processing.

##### Present-Centered Group Therapy

This therapy was initially developed by Schnurr et al. (7) to represent all of the elements of effective PTSD treatment that are not specifically trauma-focused. PCGT is a group intervention derived from and with the same content as an individual therapy (PCT) that has considerable efficacy for improvement in PTSD symptoms in military veterans (50). PCGT controls for non-specific therapeutic factors such as group bonding and therapist support, as well as contact with therapists with specialized knowledge about PTSD. It focuses on identifying and discussing current life stressors that contribute to PTSD, group problem solving, psycho-education, and promotion of wellness and physical health. Similar to the MBET group, the intervention contains daily homework assignments, involving monitoring diaries of stressful situations and when PTSD symptoms cause impairment in social and occupational functioning, and also meets for 2 h a week for 16 weeks. It does not contain instructions on mindfulness, exposure, or cognitive restructuring.

#### MRI Scanning

At week 0–1 (prior to starting group therapy) and at weeks 17–18 (after group termination), participants underwent both structural and functional MRI scanning. The functional MRI session included the emotional faces task reported here, a resting-state scan and a separate emotion regulation task in which aversive general social–emotional scenes [from the international affective picture set (“IAPS”)] were viewed (results reported separately). This paper reports results obtained from an emotional faces processing task. To control for potential order effects (“bleed over”), the order of tasks were counter balanced in a pseudo-randomized manner. It should be noted that no “trauma-related” tasks (e.g., trauma script-driven imagery, Iraq, Afghanistan, or combat-related imagery tasks, etc.) were performed in this study. fMRI data are available from *N* = 23 PTSD patients reported here, out of a total of *N* = 38 who started one of the two group therapies. Patients in the initial therapy groups in the study were not scanned.

#### fMRI Data Acquisition and Analyses

All scanning was performed using a Philips 3-Tesla MRI scanner (Phillips Medical Systems, Andover, MA, USA) in the fMRI laboratory at the Ann Arbor VA. A total of 240 T2*-weighted echo planar gradient-recall echo volumes were acquired during rest (echo time = 30 ms, repetition time = 2000 ms, 64 × 64 matrix, flip angle = 90°, field of view = 22 cm, 42 contiguous 3 mm axial slices per volume). Five additional volumes were discarded at the beginning of each run to allow for equilibration of the MRI signal. A high-resolution T1-weighted structural image (3D turbo-fast-field-echo, 1 mm isotropic voxel, 256^2^ matrix, 180 slices, repetition time = 9.8 ms, echo time = 4.6 ms, flip angle = 8°) was also obtained to provide for more precise anatomical localization. fMRI data were analyzed using the statistical parametric mapping software package, SPM8 (Wellcome Department of Cognitive Neurology, London, UK). Functional slices within each volume were sinc-interpolated, weighted in time, slice-by-slice, to correct for the sequence of slice acquisition. The functional volumes were realigned to correct for head motion and structural images coregistered to the mean of functional images. The structural images were spatially normalized to a standard Montreal Neurological Institute (MNI) template using the voxel-based morphometry toolbox (VBM8; http://dbm.neuro.uni-jena.de/vbm) and DARTEL high dimensional warping (51). Estimated deformation fields from warping were applied to normalize images to MNI space, and smoothed using an 8-mm FWHM Gaussian kernel. Functional data are detrended to account for scanner drift.

#### Experimental Paradigm

This paper reports results from a variant of an emotional faces matching task (52, 53). This task has previously been shown to reliably and robustly engage the amygdala and has been widely used to assess amygdala’s reactivity to social cues, and we have previously reported this task (54, 55). Participants viewed a trio of faces on the screen and were instructed to choose one of the two faces on the bottom that expressed the same emotion as the target face on top. The face photographs were selected from a validated stimulus set (56). The identities of the three faces were different and overall an equal number of male and female faces were presented. We studied target and congruent probe faces displaying one of three expressions (angry, fearful, or neutral); and the other (incongruent) probe faces always displayed a neutral face (during emotional target blocks) or a pseudo random emotional face (during neutral target blocks). Equal numbers of angry and fearful faces were randomly presented across trials. Blocks of face-matching tasks were interspersed with blocks of a baseline task of matching geometric shapes (circles, rectangles, and triangles) with similar instructions as above to maintain attention and provide a non-emotion processing contrast. Three blocks of each emotional face were presented, interspersed with blocks of shapes; each block was presented for 20 s.

#### Data Analysis

Data were analyzed using the statistical parametric mapping software package, SPM8 (Welcome Department of Cognitive Neurology, London, UK). Functional volumes were slice time corrected to account for temporal differences in slice acquisition time, realigned to the 10th volume to correct for head motion, segmented into gray matter, white matter, and CSF using the voxel-based morphometry toolbox (VBM8) and spatially normalized to a standard template based on the MNI reference brain using DARTEL high dimensional warping, and spatially smoothed using a 6-mm FWHM Gaussian kernel. Single-subject analysis was performed using standard GLM analysis in SPM8. Models consisted of regressors for task conditions (angry, fearful, and neutral blocks) as well as nuisance regressors consisting of the motion correction parameters from the realignment preprocessing step. Contrasts of responses of each facial expression to shapes were generated for each subject, and then entered into a second-level general linear model treating subject as a random effect (random effects analysis). We utilized the analysis strategy used by multiple previous studies (52–57) to explore the potential specific signature effects of each of the threat faces (fearful and angry) as well as neutral faces separately contrasted to the shapes condition (angry > shapes, fearful > shapes, neutral > shapes). Our approach to model each facial expression (contrasted with shapes) allows us to examine effects of treatment on each face type, and is less potentially ambiguous than using a contrast of emotional faces > neutral faces. Previous work has reported that “neutral” faces have robust amygdala responses (58–61) and amygdala responses to neutral expression faces appears to be moderate by both psychopathology (59) and facial “trait” characteristics (60, 61); and, thus, potential effects of symptoms and other moderators on both emotional and neutral faces could create ambiguity in interpreting emotional > neutral contrasts. Angry and fearful faces were also analyzed separately because of reports of different processing of angry and fearful threat cues (62), and because we are interested changes in neural responses to emotional faces over time (pre- to post-therapy). This approach represented a within-subjects, longitudinal repeated measures (pre- to post-therapy) design, in which each participant’s post-therapy scan to each face expression type (angry, fearful, and neutral) was compared to their own baseline (pre-therapy) scan of the same contrast.

In our primary analyses, we aimed to identify brain regions in which responses to emotional faces changed from pre- to post-psychotherapy in association with improvement in PTSD symptoms. We first constructed within-subjects T-maps of the Post > Pre-therapy contrasts of emotional faces > shapes to identify therapy-associated changes in brain responses. We then used a regressor of change in total CAPS scores (“deltaCAPS” regressor) in between-subjects regression analyses to identify brain activation changes that correlated with symptom change. *A priori* defined regions of interests (ROI) amygdala and mPFC/anterior cingulate were examined for differences in emotional activations, based on the published literature in PTSD neuroimaging (22–24, 63). For our primary ROI analyses, small volume correction (SVC) with family-wide error (FWE) corrected *p*-value < 0.05 was used. SVC masks were created using anatomical AAL atlas for bilateral amygdala, and mPFC. To explore direction of the changes contributing to the observed effects, signal changes were extracted from ROIs. Clusters of activation observed outside of *a priori* regions were reported as significant if they met whole-brain FWE corrected *p* < 0.05.

To identify potential differential effects of the two psychotherapies (MBET and PCGT), we conducted exploratory analyses utilizing flexible repeated-measures ANOVA (RM-ANOVA) in spm8. In this intervention outcome study, we were specifically interested in group × time interactions characterized by group differences at post-intervention due to the experimental group increasing (or decreasing) over time, but no difference at pre-intervention, and in which the control group does not change over time (known as “spreading” interactions, as opposed to “cross-over” interactions that could reflect pre-existing differences in groups at intake). To test for spreading interactions, we utilized the conjunction analysis of Friston and colleagues (64, 65) within flexible ANOVA F test maps to test the likelihood of voxels having a consistent main effect of time, group, and group × time interaction effect (each of which should exist in a consistent direction at a minimum threshold in a true spreading interaction, although each may not be individually significant) with search area restricted to areas where direction of all three effects agreed. The conjunction was performed on the F test maps for the three effects, with the search area being restricted to areas where the direction of all three effects agreed, *p*-values were adjusted for the *a priori* conditions of interest (i.e., all three signs consistent), and thresholded at *p* < 0.005.

To identify potential psychotherapy-associated changes in patterns of effective connectivity of brain regions associated with PTSD symptom improvement, we conducted exploratory analyses utilizing psychophysiological interaction (PPI) analysis. We performed PPI analyses of connectivity during processing of threat (angry faces) compared to baseline connectivity (during the shapes condition) in brain regions, associated with PTSD symptom changes. Regions with changes in activity that correlated with PTSD symptom improvement were used as “seed regions.” Seeds were 5-mm radius spheres centered around voxels showing peak correlation of post > pre-therapy T-maps with a delta CAPS regressor. De-convolved time series for each seed from each participant were multiplied by a block vector representing the contrast of interest (angry faces vs. shapes), and individual models contained regressors for the seed time series, the original conditions, and the interaction terms, and regressors were convolved with the canonical HRF (66). Resulting contrast maps were entered into second-level random effects analyses (pre- vs. post-therapy paired *t*-tests) and thresholded at *p* < 0.005.

## Results

### Participants

The two PTSD therapy groups did not significantly differ by PTSD symptom severity (CAPS scores), number of Axis I comorbidities, age, race, or medication use at intake (see Table 1).

### Effects of MBET and PCGT Group Therapy on PTSD Symptoms

**Figure 1:**
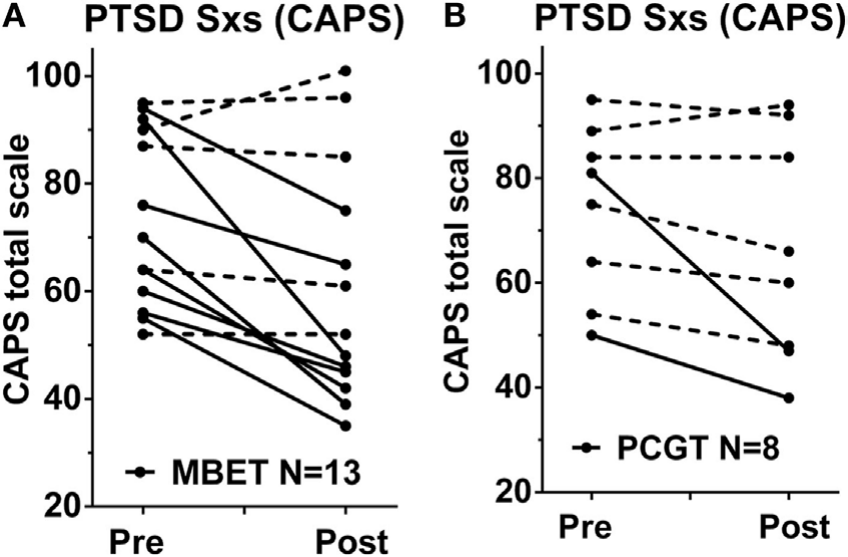
**Changes in PTSD symptoms in response to group therapy in the patients with fMRI scans in this study**. Shown are PTSD symptoms (total CAPS scores) before and after a 16-week group therapy with **(A)** mindfulness-based exposure therapy (MBET, *N* = 13 pre–post) and **(B)** present-centered group therapy (PCGT, *N* = 8 pre-post). Patients who experienced a clinically significant improvement in PTSD (change in CAPS >10 points from pre–post treatment) are shown in solid lines, patients who did not have clinically significant improvement shown in dashed lines.

The participants in this fMRI study (*N* = 23) were a subset of a larger controlled trial comparing MBET and PCGT (*N* = 38). The overall outcomes in the entire sample, as well as details of study design, compliance and retention, and detailed description of the interventions, are reported separately (King et al., unpublished data). Individual pre- and post-therapy total CAPS scores for patients in this sub-sample recruited for pre-post fMRI experiment and treated with each therapy are shown in Figure 1. MBET showed a significant reduction in total CAPS score [pre vs. post MBET *t*(13) = 3.29, *p* = 0.006, average 16 point decrease in total CAPS, effect size *d* = 0.92, effect size as per Ref. (67) Eq. 8]. PCGT was associated with a smaller decrease [pre vs. post PCGT *t*(8) = 1.81, *p* = 0.10, average 7.0 point decrease in total CAPS, *d* = 0.43]. In between condition analyses, RM-ANOVA condition × time interaction were not significant for pre–post analyses [*F*(1,20) = 1.62, *p* = 0.22] in total CAPS scores. The between condition post-therapy CAPS score effect was *d* = 0.41. Patients in the MBET group attended more group therapy sessions than patients treated with PCGT [average of 13.5 vs. 8.5 sessions, respectively, *t*(19) = 5.39, *p* < 0.001].

### Changes in Brain Responses to Emotional Faces Pre- to Post-Therapy that Correlate with Improvement in PTSD Symptoms

**Figure 2:**
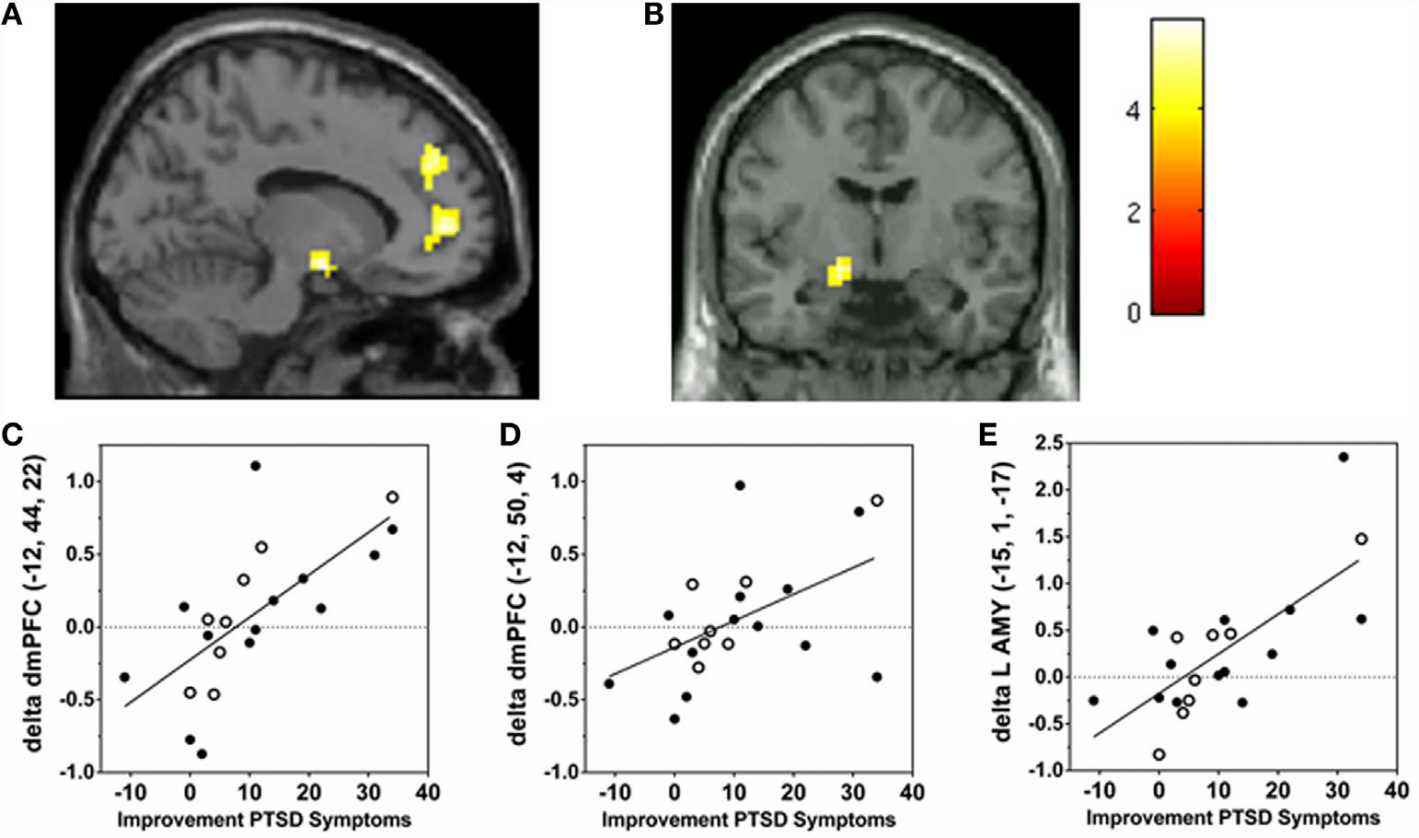
**Correlations of changes in brain responses to angry faces to improvement in PTSD symptoms pre- to post-group therapy**. Post-therapy > pre-therapy *Z*-maps of brain activations to angry faces were regressed with improvement in PTSD symptoms (change in CAPS scores) in whole-brain voxel-wise analyses (visualized at *p* < 0.001, uncorrected). **(A)** Saggital section (*x* = −12) showing dorsal medial PFC BA9 peak (−12, 44, 22) and medial PFC BA10/BA32 peak (−12, 50, 4) correlation clusters. **(B)** Coronal section (*y* = −1) showing left amygdala/peri-amygdala correlation cluster [peak (−15, −1, −17)]. **(C–E)** Scatter plots are shown to visualize distribution of improvement in PTSD vs. change in activations (betas from ROI, arbitrary units) in **(C)** dmPFC BA9, **(D)** rmPFC BA10/BA32, and **(E)** left peri-amygdala from pre- to post-therapy. Filled circles are beta values from PTSD patients treated with MBET, open circles patients treated with PCGT. (Colors of clusters represent “heat map” of *Z*-scores, see scale to right of images. All images represented in neurological convention, i.e., left side of the image corresponds to the left side of the brain).

Imaging data from two participants (one in MBET, one in PCGT condition) were excluded from the following analyses due to technical problems with data acquisition that made the data unusable. As could be expected, the emotional faces (fearful, angry, and neutral) evoked robust activations of visual cortex, dorsal medial frontal gyrus/SMA, bilateral inferior frontal gyrus, and bilateral amygdala, and de-activations of “task-negative” regions posterior cingulate and ventral medial PFC (data not shown). To test for potential order or “spill-over” effects of tasks (which were counter balanced), we examined amygdala responses in patients who had IAPS pictures or faces tasks completed first. No differences in amygdala reactions to neutral, angry, or fearful faces were observed. To identify brain changes associated with improvement in PTSD symptoms, we first searched for clusters of brain activations whose change from pre- to post-therapy was correlated with improvement in total PTSD symptoms (using a delta CAPS regressor). To maximize our power in this pilot study, we first conducted regression analyses in the total sample of PTSD patients (i.e., treated with either MBET or PCGT, *N* = 21) to identify brain regions associated with improvement in PTSD symptoms associated with either therapy. Significant clusters of correlation with PTSD symptom changes are shown in Table 2 (hypothesized regions reported as significant after SVC FWE corrected *p*-value <0.05, clusters outside of *a priori* regions reported as significant if they met whole-brain FWE corrected *p* < 0.05). Positive correlations of increased brain responses to angry faces with PTSD symptom improvement from pre- to post-therapy were identified in two regions in medial frontal cortex: a cluster spanning mPFC (BA10) and rostral ACC (BA32, Figure 2A), and a more dorsal region of medial frontal cortex spanning BA9 and BA10 (Figure 2A), and left peri-amygdala area (Figure 2B). To visualize the distribution of improvement in PTSD vs. change in activations in ROIs, scatter plots are shown in Figure 2C. dmPFC BA9, Figure 2D, rmPFC BA10/BA32, and Figure 2E, left peri-amygdala from pre- to post-therapy. All images represented in neurological convention, i.e., left side of the image corresponds to the left side of the brain. Colors of clusters represent “heat map” of *Z*-scores, see scale to right of images. No significant clusters of negative correlation were seen with the same contrast (i.e., brain responses that decreased from pre- to post-therapy correlated with PTSD symptom improvement). No significant positive or negative correlations of delta CAPS regressor with pre- to post-therapy changes in brain responses to fearful or neutral faces were detected.

**Table 2:** **Changes in brain responses to emotional faces from pre- to post-therapy that correlate with improvement in PTSD symptoms (change in total CAPS score)**.

				Peak		Cluster	
Region	*x*	*y*	*z*	*Z*-score	SVC pFWE	kE	SVC pFWE
**Angry > shape**							
**Post > pre**							
Medial frontal gyrus (BA10)/rostral ACC (BA 32)	−12	50	4	4.18	0.011	42	0.014
Medial frontal gyrus (BA9)	−9	47	37	3.56	0.078	22	0.039
(BA10)	*−12*	*44*	*22*	*3.47*	*0.100*		
Left Amygdala/peri-amygdala	−15	−1	−17	4.80	0.006	15	0.019
**Fearful > shape**							
**Post > pre**							
No significant clusters							
**Neutral > shape**							
**Post > pre**							
No significant clusters							

*Italics: peak of a subcluster*.

### Differential Changes in Responses to Emotional Faces Pre- to Post-Therapy with MBET vs. PCGT

**Figure 3:**
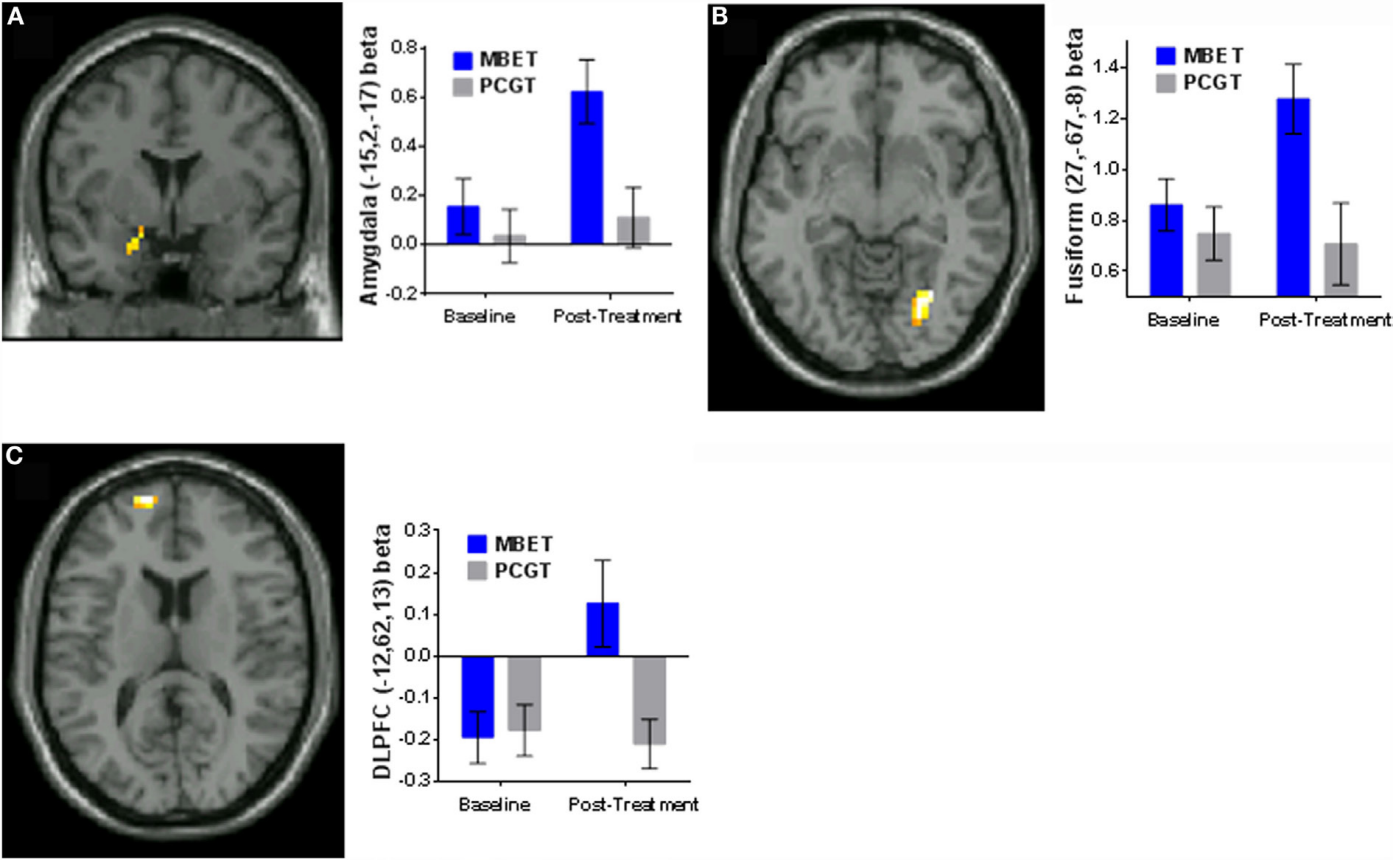
**Differential effects of MBET vs. PCGT on changes in responses to emotional faces from pre- to post-therapy (RM-ANOVA, group × time “spreading interaction”)**. **(A)** Group × time interactions in left amygdala (−15, 2, −17) in the viewing *Angry Faces* condition. **(B)** Group × time interactions in right lingual gyrus/fusiform gyrus (27, −67, −8) viewing *Angry Faces*. **(C)** Group × time interactions in left medial frontal gyrus (−12, 62, 13) viewing *Fearful Faces*.

To identify potential differential effects of therapies (MBET vs. PCGT) in evoking changes in brain responses to emotional faces related to changes in PTSD symptoms (e.g., changes in amygdala and medial frontal cortex responses), we used a whole-brain RM-ANOVA approach test group (MBET vs. PCGT) by time (pre- vs. post-therapy) interaction effects, utilizing a “spreading interaction” contrast (as described in Methods). Differential changes in brain activations to angry, fearful, and neutral faces are listed in Table 3 (group × time spreading interaction term). Treatment-specific changes in response to angry faces were observed in left amygdala, characterized by increased left amygdala responses in patients treated with MBET, but not in patients treated with PCGT (Figure 3A). Treatment-specific changes were also seen in right fusiform gyrus (Figure 3B) and left parahippocampal gyrus, as well as left precuneus, and posterior cingulate (Table 3). In responses to fearful faces, an increase in activity in an area of medial frontal cortex/BA10 was seen in MBET but not in PCGT (Table 3, Figure 3C.). In responses to neutral faces, a similar increase in response to in fusiform gyrus was seen in the MBET group only (Table 3).

**Table 3:** **Differential changes in responses to emotional faces pre- to post-therapy with MBET vs. PCGT (RM-ANOVA spreading interaction term *p* < 0.005)**.

Region	*x*	*y*	*z*	*F*	*k*
**Angry faces**					
*Clusters within search area*					
L amygdala	−15	2	−17	10.65	16
R parahippocampus	21	−10	−20	21.43	25
*Other clusters*					
R fusiform/lingual gyrus	27	−67	−8	20.80	36
R precuneus	21	−64	55	18.11	202
	*18*	−*58*	*48*	*15.75*	
Posterior cingulate	0	−46	40	10.76	15
**Fearful faces**					
*Clusters within search area*					
L medial frontal gyrus (BA10)	−12	62	13	17.19	16
*Other clusters*					
None					
**Neutral faces**					
*Clusters within search area*					
None					
*Other clusters*					
R fusiform/lingual gyrus	24	−76	−8	20.80	71
	*30*	−*68*	−*8*	*11.90*	
L lingual gyrus	−33	−78	16	12.27	73
L fusiform/lingual gyrus	−18	−70	−11	11.28	30
L caudate body	−18	−1	−13	11.12	36

*Italics: peak of a subcluster*.

### Changes in Pre- to Post-Therapy Effective Connectivity Viewing Angry Faces in Brain Regions Associated with PTSD Symptom Improvement

**Figure 4:**
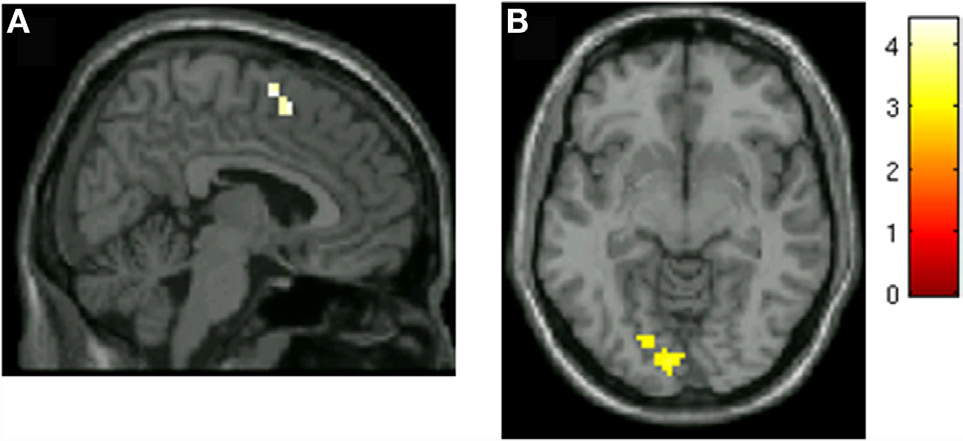
**Increased left amygdala connectivity viewing angry faces following group psychotherapy**. **(A)** Increased left amygdala connectivity with SMA/dorsal ACC. **(B)** Increased left amygdala connectivity with lingual gyrus.

Psychophysiological interaction analyses were used to identify potential changes from pre- to post-therapy in effective connectivity with the brain regions that were associated with PTSD improvement. Time series of activity of the left amygdala (−15, −1, −17) and left mPFC (−12, 50, 4) peaks of correlation with symptom improvement were used as seeds in PPI comparing viewing angry faces to baseline (viewing shapes). Table 4 shows results of changes in effective connectivity, from pre- to post-therapy Clusters of increases in effective connectivity to the left amygdala seed while viewing angry faces were seen in medial frontal gyrus (BA6)/dorsal ACC (BA 32, Figure 4A), lingual gyrus (Figure 4B), and left precentral gyrus following psychotherapy. Increased amygdala connectivity in these regions was not correlated with improvement in PTSD symptoms. No significant clusters were observed with the left mPFC seed.

**Table 4:** **Changes from pre- to post-therapy in effective connectivity viewing angry faces in brain regions associated with PTSD symptom improvement (*p* < 0.001 uncorrected)**.

Seed/region	*x*	*y*	*z*	*Z*-score	*K*
**L amygdala seed (−15, −1, −17)**					
L lingual/fusiform gyrus	−15	−76	−2	3.98	36
	*−6*	*−94*	*−5*	*3.98*	
L precentral gyrus	−39	−7	52	3.55	25
Medial frontal gyrus (BA6)/cingulate gyrus (BA32)	9	8	52	3.32	20
**L mPFC seed (−12, 50, 4)**					
No significant clusters					

*Italics: peak of a subcluster*.

## Discussion

We examined neural correlates of treatment improvement in PTSD symptoms before and after 16 weeks of MBET/PCGT group psychotherapy for PTSD in OEF/OIF veterans. We examined changes in brain responses to social–emotional cues (viewing “threat-related” faces with angry or fearful expressions) before and after two forms of group psychotherapy, a novel mindfulness-based group (MBET) and a comparison group therapy (PCGT). Improvement in PTSD symptoms irrespective of the type of group therapy was associated with increased responses in to angry faces from Pre- to Post-therapy in rostral medial PFC/peri-genual ACC (BA10/BA32); rostral/dorsal medial PFC (BA9/BA10), and left amygdala. The observed correlations in medial frontal cortex and amygdala activity likely reflect general improvement in PTSD symptoms, independent of type of therapy or treatment. We also detected evidence for potential differences in neural responses to the two treatments in the overlapping brain regions: MBET was associated with greater increases in amygdala and lingual/fusiform gyrus responses to angry faces, and an increase in medial PFC responses to fearful faces (group × time interactions).

While group psychotherapies for combat PTSD in military veterans are common in some institutions (e.g., VA hospital PTSD clinics in the USA), and even though group therapy for PTSD appears to be associated with good retention and engagement in VA settings (5), most reports to date have shown only weak efficacy for improving PTSD symptoms in military veterans and, in particular, male combat veterans (6, 7, 9). Thus, while this was a small pilot study that was primarily focused on neural correlates of group therapy for PTSD, the relatively robust effect size in the MBET condition (*d* = 0.92) suggests further study may be warranted in this modality. These findings complement other very recent studies involving group therapies for PTSD that have shown higher levels of efficacy [e.g., a controlled pilot study of group CPT in active duty military personnel (47) and an uncontrolled pilot study of group *in vivo* exposure coupled with individual imaginal trauma exposure in military veterans (68)] to suggest that some group modalities may be efficacious for military populations with PTSD. The comparison intervention used in this study (PCGT) is a group version of individual PCT that originally developed as a comparison therapy for RCTs of exposure-based PTSD therapy to control for “non-specific” therapy factors. Interestingly, although it was thought of as a “non-specific control,” PCT in the individual format has shown considerable efficacy for PTSD improvement, and in a recent meta-analysis PCT was as efficacious as exposure therapy in three out of five studies (50), with “large” effect sizes 0.77–1.27. The group form of PCT (PCGT) used here was similarly developed as a “non-specific control” for an RCT comparison to a trauma-focused group therapy (TFGT). While PCGT has shown considerably lower efficacy for PTSD than individual PCT, it should be noted that PCGT had the same effect size as TFGT (7). Versions of PCGT (adapted to be the same number of sessions as the “active” group) have also been used as a comparison intervention in recent studies of group CPT for active duty military (47) and as a comparison group MBSR in veterans with PTSD (21). Thus, although only a “medium” effect size was seen for PCGT here, it is “on par” with multiple previous reports of both trauma-focused and non-trauma-focused group therapies for PTSD in military veterans (7, 9, 21).

Presentation of human faces with “threat-related” emotional expressions have been used in a number of neuroimaging studies of psychiatric disorders, including PTSD; fearful faces have been proposed to represent indirect or ambiguous threat, and angry faces a more direct or non-ambiguous threat (69). A number of PTSD neuroimaging studies have used emotional/“threat-related” faces, as well as other cognitive-emotional tasks, and symptom-provocation (e.g., trauma scripts or trauma-related stimuli) [see meta-analyses (22–25)]. The most consistent findings in PTSD meta-analyses have been hyper-activity in mid-cingulate/dorsal ACC, insula, and amygdala, and hypo-activity in ventral medial PFC, rostral ACC, and dorsal mPFC. In the present study, we found that patients with the greatest improvement in PTSD symptoms following therapy showed greatest increases in regions of dorsal PFC BA9/BA10 previously associated with hypo-activation in PTSD. The medial PFC and rostral ACC are involved in a number of emotional regulatory processes, including effortful emotional regulation and fear extinction processes (70–73); and, thus, the observed increased activity in these regions is consistent with the notion that therapy-associated improvement in PTSD may be related to normalization of neural responses to social–emotional threat and improved emotional regulation.

However, our finding of PTSD-improvement-related increases in left amygdala activity might seem counter-intuitive in light of reports of hyper-activation of amygdala in PTSD. Exaggerated amygdala responses to threat-related emotional faces (e.g., fearful or angry faces) in PTSD have usually been found in studies using brief (200 ms) or backwards-masked presentation (<20 ms) of threat-related faces (26, 74–76); and, thus, reflect the early, relatively automatic processing of faces and related amygdala activity. However, a number of studies with longer stimulus presentation have not reported exaggerated amygdala responses to threatening stimuli in PTSD (77–80); and, furthermore, a study of explicitly presented or “unmasked” fearful faces reported a negative, rather than a positive, correlation of amygdala responses with PTSD symptoms (75).

It was furthermore interesting that changes in amygdala responses related to PTSD symptom improvements were found in angry faces, but not in fearful faces, which as described above have been used in several previous neuroimaging studies of PTSD (26, 74–76), or in neutral faces. It is of course possible that the lack of detection is due to the relatively small N of this pilot study. However, differences in fearful vs. angry facial processing has been reported in previous studies (54, 62), suggesting the intriguing possibility that changes in processing of angry faces (overt social threat) might reflect specific changes in PTSD symptoms occurring over the course of treatment. We previously reported an effect of childhood poverty on connectivity of amygdala with medial frontal cortex in angry faces but not fearful faces (54). Furthermore, a recent EEG study of threat-related faces in OEF/OIF combat veterans with and without PTSD (using a similar emotional face-matching task) similarly reported that veterans with PTSD “blunted processing” (smaller late positive potentials) and decreased accuracy in identifying angry faces but not fearful faces (81). It was proposed that PTSD may be associated with diminished processing of overt social threat in conjunction with impaired perception for angry faces specifically. Such reduced processing could represent a defensive or dissociative response that could be related to avoidant and numbing symptoms, and difficulty in engaging emotionally with trauma- or threat-related material in PTSD (82). If this is indeed the case, it is possible that the increased amygdala activity we observed to longer (3000 ms) processing of angry faces correlated to increased PTSD symptom reduction might reflect greater engagement and less avoidance of threat-related cues, and, thus, greater emotional processing of these cues. Indeed, PTSD patients who are successfully treated may be better able to separate true threat and, thus, react more robustly when confronted with actually threatening stimuli in the environment. This is also supported by the finding of greater post-therapy amygdala connectivity with visual processing areas, including lingual gyrus and fusiform gyrus, involved in visual processing of faces, although these changes in connectivity were not correlated with PTSD symptom reduction.

While the observed increased medial PFC and amygdala responses may reflect general symptom improvement, we also found evidence of potential specific effects of MBET. Group × time interactions found MBET was associated with greater post-therapy responses of both amygdala and fusiform/lingual gyrus to angry faces, as well as greater activation of a region of left medial PFC in response to fearful faces, suggesting that mindfulness-training may be associated with greater engagement with threat cues in PTSD patients. This would be consistent with the orientation of exposure, which involves engaging and “facing” feared stimuli, as well as mindfulness training, which involves engaging and observing both sensory phenomena and one’s emotional reactions to them in a balanced and accepting attitude, as opposed to either distraction/avoidance or suppression of emotional responses. A recent pre–post therapy fMRI study with angry and neutral faces with patients with GAD comparing MBSR to an active control found greater activation in ventrolateral PFC following MBSR (46), as well as a shift from negative to positive amygdala functional connectivity with medial cortex and rostral ACC regions similar to those seen correlated with PTSD symptom improvement in the present study. Interestingly, they also found increased activity in occipital lobe regions associated with visual processing of faces. Similar to our interpretation, the authors suggested that the positive amygdala–mPFC connectivity following MBSR in GAD may be due to greater neural processing of threat-related faces and engagement. It should be noted that MBET includes *in vivo* exposure as well as mindfulness training and, thus, either or both of these components could be contributing to the observed effects in this small pilot. In future, larger studies we plan to directly compare the effects of separate groups involving mindfulness training and *in vivo* exposure. Furthermore, we found an intriguing suggestion of a potentially unique neural signature in MBET: increased responses to fearful faces in a region of left OFC/medial frontal cortex was found in the MBET group only. In measures of rsFC in the same PTSD treatment study, MBET was also associated with an increase in positive rsFC of PCC with a similar region of left DLPFC (49), a pattern similar to that previously reported in long-term meditators (33), which has been proposed to reflect potential effects of mindfulness on volitional attention shifting and rumination. The findings presented here further implicate a potential effect of mindfulness-based interventions on PFC when processing threat, which may be involved in the salutary effects of mindfulness. In future studies we hope to identify and further elucidate the potential relationships between effects of mindfulness training as well as *in vivo* (non-trauma) exposure on symptom reduction, social threat processing, attention, and rumination in PTSD.

We also used PPI to conduct exploratory whole-brain analyses of potential changes in connectivity from pre- to post-therapy in those regions in which we observed PTSD improvement-related changes in the strength of responses to angry faces. Changes in connectivity from pre- to post-therapy were not detected in the mPFC/rACC seed, but increased post-therapy connectivity to the left amygdala seed was detected in medial frontal gyrus/dorsal ACC and lingual gyrus. While these exploratory findings should be considered preliminary, they suggest greater connectivity of amygdala with both visual processing and potential emotional regulatory circuits after treatment; this is again consistent with the notion of greater engagement and perceptual and emotional processing of overt social threat following treatment. However, it should be noted a significant correlation was not detected between symptom improvement and the observed changes in amygdala connectivity. In future work, we plan to follow up in larger samples potential mindfulness- and exposure treatment-related changes in amygdala connectivity while processing social–emotional threat, and the relationship to symptom changes.

### Limitations

This pilot pre- to post-therapy neuroimaging study of a novel therapy (MBET) is limited by the small number of PTSD patients with analyzable data (total *N* = 21), in particular in the PCGT group (*N* = 8). The discontinuation of the PCGT group decreased the number of patients available in the PCGT condition, thus decreasing power to detect potential effects of PCGT *per se*, and compare effects between the groups. However, the MBET group was reasonably powered for within-subjects analyses, given that a reliable effect size on PTSD symptoms was observed. We did not observe a statistically significant difference in pre-post effects of MBET and PCGT on PTSD symptoms in this small sample. While the degree of symptom reduction compared favorably with other studies of group psychotherapy for PTSD in military veterans, it was considerably smaller than effects reported for individual trauma-focused treatments (3), and the majority of patients in this study retained PTSD diagnosis following treatment. Thus, the present brain activation findings may not be generalizable to patients who achieve remission from PTSD, which could be associated with additional changes in brain function. The therapists in this study were not blind to the study hypotheses. While we found evidence of differential brain activation effects between MBET and PCGT, since the “dose” of therapy was different between the groups we are limited in making causal inferences regarding whether these are related to the different therapeutic modalities employed in the groups (e.g., mindfulness training and *in vivo* exposure).

### Summary

This pilot study of a novel mindfulness-based, group *in vivo* exposure therapy for combat PTSD (MBET) found improvement in PTSD symptoms from pre- to post treatment was associated with changes in brain responses to processing of angry faces, including increased activity in rostral and dorsal medial PFC, and left amygdala. While *N* was small for both groups, similar relationships were evident in both groups, suggesting that these may be a general effect related to PTSD symptom reduction. We also found evidence suggesting that the MBET group was associated with greater increases in amygdala and fusiform gyrus while processing angry faces, as well as greater increase in an area in left medial PFC when processing fearful faces, and exploratory evidence for potential treatment-related changes in amygdala connectivity. While these must be considered preliminary findings given the sample size, they provide intriguing evidence that group therapy for PTSD may produce changes in neural processing of social–emotional threat that are related to symptom reduction.

## Author Contributions

AK designed the study, obtained funding, performed study, analyzed and interpreted data, and wrote the initial draft of paper. fMRI data collection and analyses were performed by SB and RS. Clinical psychotherapy was performed by AK, KP, SR, and NG. AK, IL, SR, and NG were involved in study design and obtaining funding. IL, SB, RS, SR, NG, KP, and AK were involved in drafting the final manuscript.

## Conflict of Interest Statement

The authors declare that the research was conducted in the absence of any commercial or financial relationships that could be construed as a potential conflict of interest.

## References

[1] Fulton JJ Calhoun PS Wagner HR Schry AR Hair LP Feeling N The prevalence of posttraumatic stress disorder in operation enduring freedom/operation Iraqi freedom (OEF/OIF) veterans: a meta-analysis. J Anxiety Disord (2015) 31:98–107. 10.1016/j.janxdis.2015.02.003 25768399

[2] Bradley R Greene J Russ E Dutra L Westen D. A multidimensional meta-analysis of psychotherapy for PTSD. Am J Psychiatry (2005) 162:214–27. 10.1176/appi.ajp.162.2.214 15677582

[3] Steenkamp MM Litz BT Hoge CW Marmar CR. Psychotherapy for military-related PTSD: a review of randomized clinical trials. JAMA (2015) 314:489–500. 10.1001/jama.2015.8370 26241600

[4] Kehle-Forbes SM Meis LA Spoont MR Polusny MA. Treatment initiation and dropout from prolonged exposure and cognitive processing therapy in a VA outpatient clinic. Psychol Trauma (2016) 8(1):107–14. 10.1037/tra0000065 26121175 PMC9873270

[5] Sripada RK Bohnert KM Ganoczy D Blow FC Valenstein M Pfeiffer PN Initial group versus individual therapy for posttraumatic stress disorder and subsequent follow-up treatment adequacy. Psychol Serv (2016). 10.1037/ser0000077 27175477

[6] Schnurr PP Friedman MJ Engel CC Foa EB Shea MT Chow BK Cognitive behavioral therapy for posttraumatic stress disorder in women: a randomized controlled trial. JAMA (2007) 297:820–30. 10.1001/jama.297.8.820 17327524

[7] Schnurr PP Friedman MJ Foy DW Shea MT Hsieh FY Lavori PW Randomized trial of trauma-focused group therapy for posttraumatic stress disorder: results from a department of veterans affairs cooperative study. Arch Gen Psychiatry (2003) 60:481–9. 10.1001/archpsyc.60.5.481 12742869

[8] Castillo DT Lacefield K C’De Baca J Blankenship A Qualls C. Effectiveness of group-delivered cognitive therapy and treatment length in women veterans with PTSD. Behav Sci (2014) 4:31–41. 10.3390/bs4010031 25379266 PMC4219250

[9] Sloan DM Feinstein BA Gallagher MW Beck JG Keane TM Efficacy of group treatment for posttraumatic stress disorder symptoms: a meta-analysis. Psychol Trauma (2013) 5:176–83. 10.1037/a0026291

[10] Holzel BK Lazar SW Gard T Schuman-Olivier Z Vago DR Ott U. How does mindfulness meditation work? Proposing mechanisms of action from a conceptual and neural perspective. Perspect Psychol Sci (2011) 6:537–59. 10.1177/1745691611419671 26168376

[11] Vago DR Silbersweig DA. Self-awareness, self-regulation, and self-transcendence (S-ART): a framework for understanding the neurobiological mechanisms of mindfulness. Front Hum Neurosci (2012) 6:296. 10.3389/fnhum.2012.00296 23112770 PMC3480633

[12] Segal ZV Bieling P Young T MacQueen G Cooke R Martin L Antidepressant monotherapy vs sequential pharmacotherapy and mindfulness-based cognitive therapy, or placebo, for relapse prophylaxis in recurrent depression. Arch Gen Psychiatry (2010) 67:1256–64. 10.1001/archgenpsychiatry.2010.168 21135325 PMC3311113

[13] Eisendrath SJ Gillung E Delucchi K Mathalon DH Yang TT Satre DD A preliminary study: efficacy of mindfulness-based cognitive therapy versus sertraline as first-line treatments for major depressive disorder. Mindfulness (N Y) (2015) 6:475–82. 10.1007/s12671-014-0280-8 26085853 PMC4465797

[14] Eisendrath SJ Gillung EP Delucchi KL Chartier M Mathalon DH Sullivan JC Mindfulness-based cognitive therapy (MBCT) versus the health-enhancement program (HEP) for adults with treatment-resistant depression: a randomized control trial study protocol. BMC Complement Altern Med (2014) 14:95. 10.1186/1472-6882-14-95 24612825 PMC3995768

[15] Hoge EA Bui E Marques L Metcalf CA Morris LK Robinaugh DJ Randomized controlled trial of mindfulness meditation for generalized anxiety disorder: effects on anxiety and stress reactivity. J Clin Psychiatry (2013) 74:786–92. 10.4088/JCP.12m08083 23541163 PMC3772979

[16] Kimbrough E Magyari T Langenberg P Chesney M Berman B. Mindfulness intervention for child abuse survivors. J Clin Psychol (2010) 66:17–33. 10.1002/jclp.20624 19998425

[17] Earley MD Chesney MA Frye J Greene PA Berman B Kimbrough E. Mindfulness intervention for child abuse survivors: a 2.5-year follow-up. J Clin Psychol (2014) 70:933–41. 10.1002/jclp.22102 24844944

[18] Kearney DJ McDermott K Malte C Martinez M Simpson TL. Association of participation in a mindfulness program with measures of PTSD, depression and quality of life in a veteran sample. J Clin Psychol (2012) 68:101–16. 10.1002/jclp.20853 22125187

[19] Kearney DJ McDermott K Malte C Martinez M Simpson TL. Effects of participation in a mindfulness program for veterans with posttraumatic stress disorder: a randomized controlled pilot study. J Clin Psychol (2013) 69:14–27. 10.1002/jclp.21911 22930491

[20] King AP Erickson TM Giardino ND Favorite T Rauch SA Robinson E A pilot study of group mindfulness-based cognitive therapy (MBCT) for combat veterans with posttraumatic stress disorder (PTSD). Depress Anxiety (2013) 30:638–45. 10.1002/da.22104 23596092 PMC4373594

[21] Polusny MA Erbes CR Thuras P Moran A Lamberty GJ Collins RC Mindfulness-based stress reduction for posttraumatic stress disorder among veterans: a randomized clinical trial. JAMA (2015) 314:456–65. 10.1001/jama.2015.8361 26241597

[22] Hayes JP Hayes SM Mikedis AM. Quantitative meta-analysis of neural activity in posttraumatic stress disorder. Biol Mood Anxiety Disord (2012) 2:9. 10.1186/2045-5380-2-9 22738125 PMC3430553

[23] Etkin A Wager TD. Functional neuroimaging of anxiety: a meta-analysis of emotional processing in PTSD, social anxiety disorder, and specific phobia. Am J Psychiatry (2007) 164:1476–88. 10.1176/appi.ajp.2007.07030504 17898336 PMC3318959

[24] Patel R Spreng RN Shin LM Girard TA. Neurocircuitry models of posttraumatic stress disorder and beyond: a meta-analysis of functional neuroimaging studies. Neurosci Biobehav Rev (2012) 36:2130–42. 10.1016/j.neubiorev.2012.06.003 22766141

[25] Stark EA Parsons CE Van Hartevelt TJ Charquero-Ballester M McManners H Ehlers A Post-traumatic stress influences the brain even in the absence of symptoms: a systematic, quantitative meta-analysis of neuroimaging studies. Neurosci Biobehav Rev (2015) 56:207–21. 10.1016/j.neubiorev.2015.07.007 26192104

[26] Bryant RA Felmingham K Kemp A Das P Hughes G Peduto A Amygdala and ventral anterior cingulate activation predicts treatment response to cognitive behaviour therapy for post-traumatic stress disorder. Psychol Med (2008) 38:555–61. 10.1017/S0033291707002231 18005496

[27] Cisler JM Sigel BA Kramer TL Smitherman S Vanderzee K Pemberton J Amygdala response predicts trajectory of symptom reduction during trauma-focused cognitive-behavioral therapy among adolescent girls with PTSD. J Psychiatr Res (2015) 71:33–40. 10.1016/j.jpsychires.2015.09.011 26522869 PMC4826076

[28] Falconer E Allen A Felmingham KL Williams LM Bryant RA. Inhibitory neural activity predicts response to cognitive-behavioral therapy for posttraumatic stress disorder. J Clin Psychiatry (2013) 74:895–901. 10.4088/JCP.12m08020 24107763

[29] Aupperle RL Allard CB Simmons AN Flagan T Thorp SR Norman SB Neural responses during emotional processing before and after cognitive trauma therapy for battered women. Psychiatry Res (2013) 214:48–55. 10.1016/j.pscychresns.2013.05.001 23916537

[30] Simmons AN Norman SB Spadoni AD Strigo IA. Neurosubstrates of remission following prolonged exposure therapy in veterans with posttraumatic stress disorder. Psychother Psychosom (2013) 82:382–9. 10.1159/000348867 24061484

[31] Thomaes K Dorrepaal E Draijer N de Ruiter MB Elzinga BM van Balkom AJ Treatment effects on insular and anterior cingulate cortex activation during classic and emotional stroop interference in child abuse-related complex post-traumatic stress disorder. Psychol Med (2012) 42:2337–49. 10.1017/S0033291712000499 22436595

[32] van Rooij SJ Geuze E Kennis M Rademaker AR Vink M. Neural correlates of inhibition and contextual cue processing related to treatment response in PTSD. Neuropsychopharmacology (2015) 40:667–75. 10.1038/npp.2014.220 25154707 PMC4289955

[33] Brewer JA Worhunsky PD Gray JR Tang YY Weber J Kober H. Meditation experience is associated with differences in default mode network activity and connectivity. Proc Natl Acad Sci U S A (2011) 108:20254–9. 10.1073/pnas.1112029108 22114193 PMC3250176

[34] Hasenkamp W Barsalou LW. Effects of meditation experience on functional connectivity of distributed brain networks. Front Hum Neurosci (2012) 6:38. 10.3389/fnhum.2012.00038 22403536 PMC3290768

[35] Garrison KA Zeffiro TA Scheinost D Constable RT Brewer JA. Meditation leads to reduced default mode network activity beyond an active task. Cogn Affect Behav Neurosci (2015) 15:712–20. 10.3758/s13415-015-0358-3 25904238 PMC4529365

[36] Froeliger B Garland EL Kozink RV Modlin LA Chen NK McClernon FJ Meditation-state functional connectivity (msFC): strengthening of the dorsal attention network and beyond. Evid Based Complement Alternat Med (2012) 2012:680407. 10.1155/2012/680407 22536289 PMC3320106

[37] Kilpatrick LA Suyenobu BY Smith SR Bueller JA Goodman T Creswell JD Impact of mindfulness-based stress reduction training on intrinsic brain connectivity. Neuroimage (2011) 56:290–8. 10.1016/j.neuroimage.2011.02.034 21334442 PMC3072791

[38] Haase L May AC Falahpour M Isakovic S Simmons AN Hickman SD A pilot study investigating changes in neural processing after mindfulness training in elite athletes. Front Behav Neurosci (2015) 9:229 10.3389/fnbeh.2015.00229 26379521 PMC4550788

[39] Roland LT Lenze EJ Hardin FM Kallogjeri D Nicklaus J Wineland AM Effects of mindfulness based stress reduction therapy on subjective bother and neural connectivity in chronic tinnitus. Otolaryngol Head Neck Surg (2015) 152:919–26. 10.1177/0194599815571556 25715350 PMC4650869

[40] Wells RE Yeh GY Kerr CE Wolkin J Davis RB Tan Y Meditation’s impact on default mode network and hippocampus in mild cognitive impairment: a pilot study. Neurosci Lett (2013) 556:15–9. 10.1016/j.neulet.2013.10.001 24120430 PMC4022038

[41] Taren AA Gianaros PJ Greco CM Lindsay EK Fairgrieve A Brown KW Mindfulness meditation training alters stress-related amygdala resting state functional connectivity: a randomized controlled trial. Soc Cogn Affect Neurosci (2015) 10(12):1758–68. 10.1093/scan/nsv066 26048176 PMC4666115

[42] Farb NA Segal ZV Anderson AK. Mindfulness meditation training alters cortical representations of interoceptive attention. Soc Cogn Affect Neurosci (2013) 8:15–26. 10.1093/scan/nss066 22689216 PMC3541492

[43] Farb NA Segal ZV Mayberg H Bean J McKeon D Fatima Z Attending to the present: mindfulness meditation reveals distinct neural modes of self-reference. Soc Cogn Affect Neurosci (2007) 2:313–22. 10.1093/scan/nsm030 18985137 PMC2566754

[44] Goldin P Ziv M Jazaieri H Gross JJ. Randomized controlled trial of mindfulness-based stress reduction versus aerobic exercise: effects on the self-referential brain network in social anxiety disorder. Front Hum Neurosci (2012) 6:295. 10.3389/fnhum.2012.00295 23133411 PMC3488800

[45] Goldin P Ziv M Jazaieri H Hahn K Gross JJ. MBSR vs aerobic exercise in social anxiety: fMRI of emotion regulation of negative self-beliefs. Soc Cogn Affect Neurosci (2013) 8:65–72. 10.1093/scan/nss054 22586252 PMC3541489

[46] Holzel BK Hoge EA Greve DN Gard T Creswell JD Brown KW Neural mechanisms of symptom improvements in generalized anxiety disorder following mindfulness training. Neuroimage Clin (2013) 2:448–58. 10.1016/j.nicl.2013.03.011 24179799 PMC3777795

[47] Resick PA Wachen JS Mintz J Young-McCaughan S Roache JD Borah AM A randomized clinical trial of group cognitive processing therapy compared with group present-centered therapy for PTSD among active duty military personnel. J Consult Clin Psychol (2015) 83:1058–68. 10.1037/ccp0000016 25939018

[48] King AP Favorite TK Mindfulness-based cognitive therapy (MBCT) for combat-related posttraumatic stress disorder (PTSD) in mindfulness-based cognitive therapy. In: Eisendrath SJ, editor. Innovative Applications. Berlin, Germany: Springer (2016). p. 163–89.

[49] King AP Block SR Sripada RK Rauch S Giardino N Favorite T Altered default mode network (Dmn) resting state functional connectivity following a mindfulness-based exposure therapy for posttraumatic stress disorder (Ptsd) in combat Veterans of Afghanistan and Iraq. Depress Anxiety (2016) 33:289–99. 10.1002/da.22481 27038410

[50] Frost ND Laska KM Wampold BE. The evidence for present-centered therapy as a treatment for posttraumatic stress disorder. J Trauma Stress (2014) 27:1–8. 10.1002/jts.21881 24515534

[51] Ashburner J A fast diffeomorphic image registration algorithm. Neuroimage (2007) 38:95–113. 10.1016/j.neuroimage.2007.07.007 17761438

[52] Hariri AR Mattay VS Tessitore A Fera F Weinberger DR. Neocortical modulation of the amygdala response to fearful stimuli. Biol Psychiatry (2003) 53:494–501. 10.1016/S0006-3223(02)01786-9 12644354

[53] Hariri AR Tessitore A Mattay VS Fera F Weinberger DR. The amygdala response to emotional stimuli: a comparison of faces and scenes. Neuroimage (2002) 17:317–23. 10.1006/nimg.2002.1179 12482086

[54] Javanbakht A King AP Evans GW Swain JE Angstadt M Phan KL Childhood poverty predicts adult amygdala and frontal activity and connectivity in response to emotional faces. Front Behav Neurosci (2015) 9:154. 10.3389/fnbeh.2015.00154 26124712 PMC4464202

[55] Evans GW Swain JE King AP Wang X Javanbakht A Ho SS Childhood cumulative risk exposure and adult amygdala volume and function. J Neurosci Res (2016) 94(6):535–43. 10.1002/jnr.23681 26469872 PMC4833698

[56] Gur RC Schroeder L Turner T McGrath C Chan RM Turetsky BI Brain activation during facial emotion processing. Neuroimage (2002) 16(3):651–62. 10.1006/nimg.2002.1097 12169250

[57] Salgado-Pineda P Fakra E Delaveau P Hariri AR Blin O. Differential patterns of initial and sustained responses in amygdala and cortical regions to emotional stimuli in schizophrenia patients and healthy participants. J Psychiatry Neurosci (2010) 35:41–8. 10.1503/jpn.090017 20040245 PMC2799503

[58] Blasi G Hariri AR Alce G Taurisano P Sambataro F Das S Preferential amygdala reactivity to the negative assessment of neutral faces. Biol Psychiatry (2009) 66:847–53. 10.1016/j.biopsych.2009.06.017 19709644 PMC3013358

[59] Cooney RE Atlas LY Joormann J Eugene F Gotlib IH. Amygdala activation in the processing of neutral faces in social anxiety disorder: is neutral really neutral? Psychiatry Res (2006) 148:55–9. 10.1016/j.pscychresns.2006.05.003 17030117

[60] Said CP Sebe N Todorov A. Structural resemblance to emotional expressions predicts evaluation of emotionally neutral faces. Emotion (2009) 9:260–4. 10.1037/a0014681 19348537

[61] Todorov A Engell AD. The role of the amygdala in implicit evaluation of emotionally neutral faces. Soc Cogn Affect Neurosci (2008) 3:303–12. 10.1093/scan/nsn033 19015082 PMC2607057

[62] Whalen PJ Shin LM McInerney SC Fischer H Wright CI Rauch SL. A functional MRI study of human amygdala responses to facial expressions of fear versus anger. Emotion (2001) 1:70–83. 10.1037/1528-3542.1.1.70 12894812

[63] Simmons AN Matthews SC. Neural circuitry of PTSD with or without mild traumatic brain injury: a meta-analysis. Neuropharmacology (2012) 62:598–606. 10.1016/j.neuropharm.2011.03.016 21420986

[64] Friston KJ Holmes AP Price CJ Buchel C Worsley KJ. Multisubject fMRI studies and conjunction analyses. Neuroimage (1999) 10:385–96. 10.1006/nimg.1999.0484 10493897

[65] Friston KJ Penny WD Glaser DE. Conjunction revisited. Neuroimage (2005) 25:661–7. 10.1016/j.neuroimage.2005.01.013 15808967

[66] McLaren DG Ries ML Xu G Johnson SC. A generalized form of context-dependent psychophysiological interactions (gPPI): a comparison to standard approaches. Neuroimage (2012) 61:1277–86. 10.1016/j.neuroimage.2012.03.068 22484411 PMC3376181

[67] Morris SB DeShon RP. Combining effect size estimates in meta-analysis with repeated measures and independent-groups designs. Psychol Methods (2002) 7:105–25. 10.1037/1082-989X.7.1.105 11928886

[68] Smith ER Porter KE Messina MG Beyer JA Defever ME Foa EB Prolonged exposure for PTSD in a Veteran group: a pilot effectiveness study. J Anxiety Disord (2015) 30:23–7. 10.1016/j.janxdis.2014.12.008 25594370

[69] Kim MJ Loucks RA Palmer AL Brown AC Solomon KM Marchante AN The structural and functional connectivity of the amygdala: from normal emotion to pathological anxiety. Behav Brain Res (2011) 223:403–10. 10.1016/j.bbr.2011.04.025 21536077 PMC3119771

[70] Duval ER Javanbakht A Liberzon I. Neural circuits in anxiety and stress disorders: a focused review. Ther Clin Risk Manag (2015) 11:115–26. 10.2147/TCRM.S48528 25670901 PMC4315464

[71] Liberzon I Sripada CS. The functional neuroanatomy of PTSD: a critical review. Prog Brain Res (2008) 167:151–69. 10.1016/S0079-6123(07)67011-3 18037013

[72] Maren S Phan KL Liberzon I. The contextual brain: implications for fear conditioning, extinction and psychopathology. Nat Rev Neurosci (2013) 14:417–28. 10.1038/nrn3492 23635870 PMC5072129

[73] Shin LM Liberzon I. The neurocircuitry of fear, stress, and anxiety disorders. Neuropsychopharmacology (2010) 35:169–91. 10.1038/npp.2009.83 19625997 PMC3055419

[74] Rauch SL Whalen PJ Shin LM McInerney SC Macklin ML Lasko NB Exaggerated amygdala response to masked facial stimuli in posttraumatic stress disorder: a functional MRI study. Biol Psychiatry (2000) 47:769–76. 10.1016/S0006-3223(00)00828-3 10812035

[75] Armony JL Corbo V Clement MH Brunet A. Amygdala response in patients with acute PTSD to masked and unmasked emotional facial expressions. Am J Psychiatry (2005) 162:1961–3. 10.1176/appi.ajp.162.10.1961 16199845

[76] Shin LM Wright CI Cannistraro PA Wedig MM McMullin K Martis B A functional magnetic resonance imaging study of amygdala and medial prefrontal cortex responses to overtly presented fearful faces in posttraumatic stress disorder. Arch Gen Psychiatry (2005) 62:273–81. 10.1001/archpsyc.62.3.273 15753240

[77] Bremner JD Narayan M Staib LH Southwick SM McGlashan T Charney DS. Neural correlates of memories of childhood sexual abuse in women with and without posttraumatic stress disorder. Am J Psychiatry (1999) 156:1787–95. 10553744 10.1176/ajp.156.11.1787 PMC3233772

[78] Britton JC Phan KL Taylor SF Fig LM Liberzon I. Corticolimbic blood flow in posttraumatic stress disorder during script-driven imagery. Biol Psychiatry (2005) 57:832–40. 10.1016/j.biopsych.2004.12.025 15820703

[79] Lanius RA Williamson PC Boksman K Densmore M Gupta M Neufeld RW Brain activation during script-driven imagery induced dissociative responses in PTSD: a functional magnetic resonance imaging investigation. Biol Psychiatry (2002) 52:305–11. 10.1016/S0006-3223(02)01367-7 12208637

[80] Phan KL Britton JC Taylor SF Fig LM Liberzon I. Corticolimbic blood flow during nontraumatic emotional processing in posttraumatic stress disorder. Arch Gen Psychiatry (2006) 63:184–92. 10.1001/archpsyc.63.2.184 16461862

[81] MacNamara A Post D Kennedy AE Rabinak CA Phan KL. Electrocortical processing of social signals of threat in combat-related post-traumatic stress disorder. Biol Psychol (2013) 94:441–9. 10.1016/j.biopsycho.2013.08.009 24025760

[82] Foa EB Hearst-lkeda D Emotional dissociation in response to trauma: an information processing approach. In: Michelson LK Ray WJ, editors. Handbook of Dissociation: Theoretical, Empirical, and Research Perspectives. New York: Plenum (1996). p. 207–26.

